# Psychedelics as a Treatment for Alzheimer’s Disease Dementia

**DOI:** 10.3389/fnsyn.2020.00034

**Published:** 2020-08-21

**Authors:** Simon Andrew Vann Jones, Allison O’Kelly

**Affiliations:** Cornwall Partnership NHS Foundation Trust, Liskeard, United Kingdom

**Keywords:** psychedelic, Alzheimer’s disease, dementia, plasticicity, microdosing

## Abstract

Currently, there are no disease-modifying treatments for Alzheimer’s disease (AD) or any other dementia subtype. The renaissance in psychedelic research in recent years, in particular studies involving psilocybin and lysergic acid diethylamide (LSD), coupled with anecdotal reports of cognitive benefits from micro-dosing, suggests that they may have a therapeutic role in a range of psychiatric and neurological conditions due to their potential to stimulate neurogenesis, provoke neuroplastic changes and reduce neuroinflammation. This inevitably makes them interesting candidates for therapeutics in dementia. This mini-review will look at the basic science and current clinical evidence for the role of psychedelics in treating dementia, especially early AD, with a particular focus on micro-dosing of the classical psychedelics LSD and psilocybin.

## Introduction

Globally an estimated 50 million people have a diagnosis of dementia and population prevalence continues to increase (Alzheimer’s Society, 2019; UK Government Web Archive, 2020). Alzheimer’s disease (AD) accounts for approximately 50–70% of cases (Draper, 2013).

AD is a progressive neurological disorder characterized by extracellular amyloid protein deposition and intracellular tau protein aggregates (tangles) that, in accumulation, are associated with a variety of pathological processes including microtubular damage, axonal transport disruption and, ultimately, cell death. The hippocampus, a key structure in the ability to learn and retain information and a site for neurogenesis, is particularly vulnerable to AD pathology and one of the earliest parts of the brain to be affected by the disease (Mu and Gage, 2011; Pilly and Grossberg, 2012; Setti et al., 2017).

Currently, there is a renaissance of research using psychedelics, potent 5HT2A receptor (5HT2A-R) agonists, in psychiatric and neurological disorders (Nichols, 2004; Halberstadt, 2015; Aday et al., 2020). The 5HT2A-R is found in high concentrations in regions of the brain vulnerable to dementia such as the prefrontal cortex and aforementioned hippocampus (Wood et al., 2012; Catlow et al., 2013; Bryson et al., 2017). Psychedelics induce brain plasticity and modify connectivity between brain regions and there is considerable anecdotal evidence of cognitive benefits from micro-dosing—a dose that does not cause perceptual change or impair functioning (Carhart-Harris et al., 2012; Wood et al., 2012; Catlow et al., 2013; Muthukumaraswamy et al., 2013; Zhang and Stackman, 2015; Griffiths et al., 2016; Carhart-Harris and Goodwin, 2017a; Carhart-Harris and Nutt, 2017; Johnstad, 2018; Fadiman and Korb, 2019; Kuypers et al., 2019; Lea et al., 2020a).

This mini-review will explore the role of classical psychedelics psilocybin and Lysergic acid diethylamide (LSD) in treating AD with a focus on sub-perceptual- or “micro”- dosing. Promoting neuroplasticity and neurogenesis *via* the 5HT2A-R in regions such as the hippocampus could theoretically help protect this and other brain structures and may, therefore, hold potential for treating AD.

## Cognitive Effects

High dose psilocybin reduces attention to both clinical and electrophysiological parameters. However, this may be due to an increased awareness of sensory stimuli that are usually filtered out (cost of attention) rather than reduced attentional ability *per se* (Carter et al., 2005; Bravermanová et al., 2018).

In younger adults, the only controlled studies of micro-dosing LSD to date (*n* = 20 and *n* = 24, respectively), both using a within-subjects design, found no effects either positive or negative on the cognitive function of healthy volunteers at different sub-perceptual doses (Bershad et al., 2019; Hutten et al., 2020). Participants had all previously experienced psychedelics. The former study used a placebo, 6.5, 13, and 26 μg, and the latter study placebo, 5, 10, or 20 μg. In the latter study, subjects had objective increases in psychomotor vigilance (and subjective happiness and mood scores) coupled with a paradoxical reduction in concentration and reduced set-shifting ability at the highest microdose (20 μg) hours after ingestion. Participants also reported subjectively greater productivity at 10 micrograms and no discernible subjective or objective differences at five micrograms compared to placebo (Hutten et al., 2020). Importantly, subjects were aware that they were on the active drug at the two higher doses and had the experience of recreational drug use.

A 2018 uncontrolled, open-label naturalistic trial found increased cognitive fluency, flexibility, and originality amongst the 33 participants at various micro-doses of psilocybin (Prochazkova et al., 2018). However, results should be interpreted with a degree of caution due to the risk of selection bias (the study was organized by the Dutch Psychedelic Society), a lack of a placebo control arm, risk of practice effect bias, and no intention-to-treat analysis.

In older adults, a recent double-blind placebo-controlled study in older adults (*n* = 48) who had not taken LSD for at least the past 5 years found no difference in the number of adverse events (including cognitive impairment) between those taking placebo, 5, 10, or 20 μg doses every 5 days for 28 days (Family et al., 2020). Headaches were reported more often in those taking LSD however the small number of participants and non-linear dose-response makes this difficult to interpret. In general, the medication was well tolerated with no serious adverse events or drop-outs.

## Longer-Term Effects

In rat models, 5HT2A-R activation with mid-dose psilocybin (0.13 mg/kg) enhances both prospective and retrospective learning with lesser effects at low-dose (0.06 mg/kg; Buchborn et al., 2014; Cini et al., 2019). Consecutive daily dosing diminished benefits, and older rodents learning was enhanced by an enriched environment (Buchborn et al., 2014).

An observational study of 89 recreational users micro-dosing psychedelics found self-reported improvement across multiple psychological domains, including creativity and attention, with a sustained improvement over 6 weeks (Polito and Stevenson, 2019). Studies of recreational micro-dosing, highly vulnerable to bias but arguably self-selecting for longer-term users, report improvement in cognitive focus and attention (14–61% of users; Anderson et al., 2019; Hutten et al., 2019; Lea et al., 2020b). However only one of these studies reported figures for the duration of use, with 60.5% of respondents using for 3 months of more (Lea et al., 2020b).

There have been no properly controlled studies of micro-dosing in cognitively impaired humans or effects on cognition or mood beyond the acute phase. However, studies of high dose LSD and psilocybin have shown long-term benefits on mood. A study of 16 healthy subjects showed subjective benefits of a single dose of 200 μg LSD 12 months later, with 10 participants rating the experience as one of the top 10 most meaningful of their lives (Schmid and Liechti, 2018). In 10 patients with a life-threatening disease, LSD-assisted psychotherapy reduced anxiety significantly, an effect that persisted 12-months after therapy in 77.7%. Two-thirds of the respondents also reported that the experience has improved their quality of life (Gasser et al., 2015).

Similar results have been observed following high dose psilocybin both in patients with anxiety related to life-threatening cancer, and cancer-related depression and anxiety (*n* = 51 and 29 respectively, both cross-over design; Griffiths et al., 2016; Ross et al., 2016). Both studies reported that approximately 60–80% of participants had a clinically significant response that was sustained some 6 months later. The latter study also followed up 4.5 years later and found that these results were sustained, with 71–100% of participants reporting the experience being one of the most meaningful of their lives (Agin-Liebes et al., 2020).

In treatment-resistant depression, 10 mg and 25 mg of psilocybin given 1 week apart (*n* = 20), led to clinical response or remission in 14 participants sustained at assessment 5 weeks later. This effect persisted at 6 months follow up despite no further treatment (Carhart-Harris et al., 2018).

These encouraging results have led, in part, to approval for a trial of high-dose psilocybin specifically targeting depression in early AD (Clinicaltrials.gov, 2020).

## Neurobiological Effects

Specific 5HT2A-R polymorphisms impair verbal memory recall and object recognition and reduced 5HT2A-R density in areas of the brain responsible for key memory processes are associated with worse cognitive performance (Schott et al., 2011). Pre-task 5HT2A-R activation in mice enhances post-task hippocampal long-term potentiation and enables the re-consolidation of fear conditioning in the amygdala supporting a critical role in neuroplasticity (Catlow et al., 2013; Zhang et al., 2013). This effect can be reproduced in rats and rabbits by very low dose psychedelics but is abolished by higher doses (Romano et al., 2010; Cameron et al., 2019).

In rats, 5HT2A-R activation stimulates neurogenesis and brain-derived neurotrophic factor (BDNF) expression in the neocortex but appears to consistently inhibit the same process in the hippocampus (Vaidya et al., 1997). This may be dose-dependent, with higher doses suppressing neurogenesis beyond a certain threshold. In cultured rat neurons, activation also stimulates dendritic spine proliferation and growth (Jones et al., 2009; Yoshida et al., 2011). In a mouse model of fear conditioning, both low and high dose psilocybin led to complete resolution of a cued fear response in animals that had been primed for a shock by an auditory tone (Catlow et al., 2013). This process was more rapid at lower doses where hippocampal neurogenesis was unimpaired. In rat cortical neuron cultures and drosophila larvae, LSD promotes neurogenesis and synaptogenesis in a dose-dependent manner suggesting both an important cross-species evolutionary pathway for this effect and that there may be an optimal dose to which it may be therapeutic for this purpose (Ly et al., 2018).

There is also likely to be an optimal dose spacing. Repeated administration of LSD and/or psilocybin leads to rapid tolerance, or tachyphylaxis, of mental effects from 24 h which peaks after just four consecutive daily doses, cannot be overcome even with substantial dose increases or switching to the other substance (cross-tolerance) and is completely reversed by 5 days of abstinence (Buchborn et al., 2016). In rats, high doses of LSD (0.16 mg/kg) given every 2 days for 90 days resulted in hyperactive and asocial symptoms (Martin et al., 2014). The aforementioned double-blind placebo-controlled safety study in older adults using a schedule of a dose every fourth day (Family et al., 2020). This may be optimal as tachyphylaxis is unlikely at this infrequency and, importantly, side effects were minimal and not significantly different to those on placebo.

## Neurophysiological Effects

Human gamma frequency oscillations (30–100 Hz) within neuronal networks are important for communication between brain regions, particularly those involving attention and memory (Jensen et al., 2007; Verret et al., 2012; Mably and Colgin, 2018). These networks become disrupted decades before the onset of symptoms in AD, possibly linked to dysfunctional inhibitory interneurons leading to the disruption of the gamma-mediated temporal structure for cortical processing which allows for the coherent packaging of sensory information (Weber and Andrade, 2010; Palop and Mucke, 2016).

Studies in mild cognitive impairment and AD show contradictory results on levels of gamma activity with some showing an increase and others a decrease in vulnerable brain regions and networks (König et al., 2005; Van Deursen et al., 2008; Basar et al., 2017; Wang et al., 2017). However, a recent study found that gamma frequency response is slowed in response to stimulus in Alzheimer patients suggesting that the increase in gamma power seen in some studies with AD patients may the greater use of brain resources to maintain resting state (Basar et al., 2016). The same researchers found that long-distance gamma-related connectivity was heightened in AD patients compared to controls (Basar et al., 2017). It is possible that this increase in gamma activity is an initial response to brain failure but that this process is fatigable.

In recent studies, enhancing gamma frequency oscillations *via* external stimuli reduced amyloid burden, possibly *via* increased microglia activity, and improved cognitive function in rodents (Laccarino et al., 2016; Martorell et al., 2019). 5HT2A-R agonists enhance the power of gamma-frequency recordings suggesting a role for the 5HT2A-R both in mediating long-range projections and reducing focal Alzheimer’s pathology (Puig et al., 2010; Athilingam et al., 2017).

## Neuroimaging

In AD there is a reduction in global brain glucose metabolism which is marked in frontal and temporal-parietal areas (Garibotto et al., 2017; Rice and Bisdas, 2017). In the only psilocybin FDG-PET study to date, in healthy volunteers, acute ingestion of a 15 mg or 20 mg dose increased global brain glucose metabolism by approximately 25%, particularly in the frontal and medial temporal cortex (Vollenweider et al., 1997).

A 2019 fMRI study showed lasting benefits 4 months after a single dose of 315 μg/kg psilocybin in a group of 38 meditators (Smigielski et al., 2019). Acute MRI changes—reduced connectivity between self-perceiving medial prefrontal and ventral cingulate areas—were associated with positive changes at 4 months. A 2020 fMRI study involving 16 patients with depression who took a single dose of 10 mg of psilocybin and 25 mg a week later, found increased functional connectivity between the ventromedial prefrontal cortex and the default mode network in responders the day following treatment completion, with changes sustained 5 weeks post-dosing (Carhart-Harris et al., 2017b). Functional connectivity was increased between regions with high 5HT2A-R density suggesting that reorganizing of dysfunctional neural circuitry is an important component of the neuroplastic effects of 5HT2A-R agonists (Tagliazucchi et al., 2016; Deco et al., 2018).

Studies suggest that at least some of the antidepressant effect from psilocybin may be mediated *via* improved top-down control of the limbic system, which holds significant promise for impulse control and well-being in dementia where this control has diminished. In healthy adults, high-dose psilocybin (16 mg/kg and) has been shown to attenuate amygdala reactivity to emotional stimuli (Kraehenmann et al., 2015). In 19 adults with treatment-resistant depression, 10 mg and 25 mg psilocybin given a week apart, improved functional connectivity between the cortex and amygdala a day after the higher dose (Mertens et al., 2020). In another study, also in healthy adults, sub-perceptual doses of LSD (13 μg) was shown to significantly influence functional connectivity between the amygdala and other key regions within the limbic system suggesting 5HT2A-R mediated reorganization of more primitive networks is possible without profound acute perceptual changes, although whether these changes were lasting is unclear (Bershad et al., 2020).

These imaging studies reveal the potent reorganization of dysfunctional brain networks in affective and anxiety states. Such changes may also yield improvements in cognition, mood, and behavior by ameliorating dysfunctional circuits in cognitive impairment and dementia.

## Anti-inflammatory Mechanisms

All known genetic and environmental risk factors for AD are associated with increased inflammation, suggesting that reducing inflammation could be a target for preventing AD (Jones and Kounatidis, 2017). Psychedelics have been shown to have potent anti-inflammatory properties and, given their affinity for the 5HT2A-R, may represent a unique anti-inflammatory overwhelmingly targeted to brain tissue (Flanagan and Nichols, 2018).

In a rodent model of AD induced by chronic intracerebral inoculation of streptozotocin, 5HT1A- and 5HT2A-R agonists had a significant independent and synergistic neuroprotective effect in hippocampal neurons at 35 days *via* anti-apoptotic pathways (Shahidi et al., 2019). This neuroprotection infers activation of anti-inflammatory pathways, the corollary to this being that activation of 5HT2A-R in rodent neurons protects against reactive oxygen species (ROS) *via* the upregulation of neuroprotective Sirtuin 1 expression (Fanibunda et al., 2019). This pathway simultaneously stimulates mitochondrial biogenesis leading to greater availability of adenosine triphosphate and suggesting the potential for psychedelics to address impaired energy metabolism, another key pathological pathway to cognitive dysfunction in AD (Kapogiannis and Mattson, 2011).

## Discussion

After decades of repeated failure of treatments for dementia, there is an urgent need to develop new treatments for AD. The potential for psychedelic compounds to influence and enhance functional neuronal connectivity, stimulate neurogenesis, restore brain plasticity, reduce inflammation and enhance cognition provides a new therapeutic target and compelling argument for further investigation of the potential for psychedelics as a disease-modifying compound in conditions where currently none exists.

Animal models testing the neurobiological effects of psychedelic compounds have demonstrated hippocampal neurogenesis at lower doses and suppression at higher doses and potent neuroprotective properties. Studies in people suffering from depression and anxiety disorders have demonstrated lasting neuroplastic changes following just one or two large doses. This suggests a potential role for both sub-perceptual “micro”- and psychedelic-doses as a strategy for neuroprotection and cognitive enhancement in prodromal AD. For cognitive enhancement, the ideal dose and frequency have yet to be determined however the rapid desensitization of 5HT2A receptors by both psilocybin and LSD suggests that daily dosing is unlikely to be the optimal strategy.

Despite anecdotal evidence of widespread recreational use of micro-dosing for cognitive enhancement, robust scientific studies of the cognitive effects of micro-dosing in humans have so far been limited to acute changes in very small studies in cognitively normal individuals with no reports of persistent cognitive changes, either positive or negative, at psychedelic doses. Studies looking at both micro-dosing and psychedelic doses, longer-term, in cognitively impaired individuals are lacking and urgently needed.

## Author Contributions

SV came up with the idea for the review. Both SV and AO’K were involved in a review of the literature and writing and editing the final manuscript.

## Conflict of Interest

The authors declare that the research was conducted in the absence of any commercial or financial relationships that could be construed as a potential conflict of interest.
