# Corrigendum: Psychedelics as a Treatment for Alzheimer's Disease Dementia

**DOI:** 10.3389/fnsyn.2020.607194

**Published:** 2020-10-26

**Authors:** Simon Andrew Vann Jones, Allison O'Kelly

**Affiliations:** Cornwall Partnership NHS Foundation Trust, Liskeard, United Kingdom

**Keywords:** psychedelic, Alzheimer's disease, dementia, plasticicity, microdosing

In the original article, there was an error. In the “Neurophysiological effects” section, second paragraph, there was an error message in between two of the references: “Error! Bookmark not defined”. All the references are present and correct.

A correction has been made to the “Neurophysiological effects” section, second paragraph. The sentence “Error! Bookmark not defined” has been removed.

The authors apologize for this error and state that this does not change the scientific conclusions of the article in any way. The original article has been updated.

